# Fall risk assessment using the World Guidelines for Falls Prevention Algorithm: Evidence from the ELSI-Brazil study

**DOI:** 10.1007/s41999-026-01446-6

**Published:** 2026-04-01

**Authors:** Núbia Carelli Pereira de Avelar, Ana Lúcia Danielewicz, Bruno de Souza Moreira, Amanda Cristina de Souza Andrade, Juliana Lustosa Torres, Juleimar Soares Coelho de Amorim, Manuel Montero-Odasso, Maria Fernanda Lima-Costa, Monica Rodrigues Perracini

**Affiliations:** 1 Laboratory of Aging, Resources and Rheumatology, Department of Physiotherapy, Federal University of Santa Catarina, Campus Araranguá, Rod. Governador Jorge Lacerda, 3201, Urussanguinha, Araranguá, Santa Catarina 88906-072 Brazil; 2 Center for Studies in Public Health and Aging, Federal University of Minas Gerais, Belo Horizonte, Brazil; 3 René Rachou Institute, Oswaldo Cruz Foundation - Minas Gerais, Belo Horizonte, Minas Gerais Brazil; 4 Department of Preventive and Social Medicine, Federal University of Minas Gerais, Belo Horizonte, Minas Gerais Brazil; 5 Postgraduate Program in Public Health, Federal University of Minas Gerais, Belo Horizonte, Minas Gerais Brazil; 6 Physical Therapy Course, Federal Institute of Rio de Janeiro, Rio de Janeiro, Brazil; 7 Schulich School of Medicine and Dentistry, Western University, London, ON Canada; 8 Division of Geriatric Medicine, Parkwood Institute and Lawson Research Institute, London, ON Canada; 9 Master′s and Doctoral Programs in Physical Therapy, Universidade Cidade de São Paulo, São Paulo, Brazil; 10 Master′s and Doctoral Programs in Gerontology, Universidade Estadual de Campinas, São Paulo, Brazil

**Keywords:** Accidental falls, Community-dwelling, Older adults, Prevalence

## Abstract

**Background:**

The World Guidelines for Falls Prevention and Management (WGF) propose a global algorithm to stratify fall risk in older adults using fall history (FH) or key questions (KQ). However, evidence on the applicability of this screening tool in low- and middle-income countries, and on whether these approaches produce different population estimates, remains limited. Implementing this tool may enhance the identification of older adults at higher risk of falls.

**Objective:**

This study estimated national and regional fall risk prevalence in Brazil using both methods and examined geographic differences.

**Methods:**

We conducted a cross-sectional study using data from older participants (≥ 60 years) of the third wave of the Brazilian Longitudinal Study of Aging (ELSI-Brazil, 2023–2024), a nationally representative study. According to the WGF algorithm, participants were classified as high, intermediate, or low fall risk based on FH or two KQ (concern about falling and postural instability) combined with gait speed ≤ 0.8 m/s and severity markers. KQ included concern about falling, assessed using the short version of the Falls Efficacy Scale-International, and postural instability evaluated using the tandem stance test (impaired balance if < 10 s). Poisson regression with robust variance adjusted for sociodemographic factors was used to examine regional differences in fall risk prevalence.

**Results:**

A total of 7515 older adults participated in this study (67.8 ± 0.2 years; 57.1% female). Using FH alone, 82.2% of older adults were classified as low risk, 7.8% as intermediate, and 10.0% as high risk. Using KQ, the prevalence was 50.4%, 34.6%, and 15.0%, respectively. With the KQ approach, older adults in the Northeast and Southeast had a lower probability of being low risk and a higher probability of being intermediate risk compared with those in the South. No regional differences were observed for high-risk classification or when FH alone was used.

**Conclusion:**

Operationalizing the WGF algorithm with KQ substantially increases intermediate fall risk identification compared to FH alone. Based on national population estimates, approximately 3.5 million older Brazilian adults may be at high risk of falls. Within-country regional disparities reinforce the need for tailored strategies to address contextual differences in fall prevention.

**Supplementary Information:**

The online version contains supplementary material available at 10.1007/s41999-026-01446-6.

## Key summary points

**Aim:**

To examine how applying the World Guidelines for Falls Prevention algorithm, using fall history alone versus key screening questions, affects fall-risk stratification in older Brazilian adults.

**Findings:**

Using key screening questions more than quadrupled the proportion classified as intermediate and revealed regional differences, with higher intermediate risk in the Northeast and Southeast.

**Message:**

Incorporating key screening questions improves risk detection and highlights regional inequities, supporting their routine use in fall-risk assessment in Brazil.

**Supplementary Information:**

The online version contains supplementary material available at 10.1007/s41999-026-01446-6.

## Introduction

Falls are a highly prevalent event among the older population. A systematic review indicated a global prevalence of 26.5% [1], a pattern similar to that observed in a meta-analysis of Brazilian studies (27.0%) [2]. According to the 2023 Global Burden of Disease estimates, falls ranked 17th among 292 causes of death [3], and projections indicate that fall-related deaths will continue to increase until 2040 [4].

Screening and stratifying fall risk are essential for healthcare services to implement targeted interventions and effective strategies to prevent future incidents. Early detection is crucial, as many older adults do not seek healthcare services after a fall and may hesitate to report the event during routine consultations. In this context, algorithms have been proposed to facilitate the early identification of individuals at increased risk of falling [5, 6]. The World Guidelines for Falls Prevention and Management for Older Adults (WGF) is the most recent proposal for classifying fall risk in older adults and recommends considering the fall history (FH) in the past 12 months plus key questions (KQ) that address aspects such as FH, concern about falling to improve accuracy, and subjective instability in the orthostatic position or during gait [6].

Although the algorithm recommends using KQ to increase sensitivity in the early detection of individuals at risk of falling, to date, only one study has evaluated the impact of this inclusion in older adults [7]. van de Loo et al. (2024) demonstrated that relying solely on FH underestimates the number of Dutch individuals at intermediate fall risk [7]. Identifying this intermediate group is an important distinguishing feature of the WGF algorithm compared to other algorithms [5]. Moreover, other studies suggest that the WGF algorithm has a limited ability to distinguish between individuals at low and intermediate fall risk [8–10]. The uncertainty about stratification raises questions about the actual distinction between low- and intermediate-risk groups, as well as the recommended clinical applicability of screening and intervention. Given this context, further studies are needed to assess whether different WGF approaches affect fall risk prevalence among older adults. A more accurate identification of risk levels could improve the selection of groups for more effective clinical interventions, optimize resource use, and strengthen prevention strategies, ensuring that older adults at higher risk receive timely and appropriate care. This aspect is particularly relevant in low- and middle-income countries (LMICs), like Brazil, where resources are more limited.

Globally, most older adults live in LMICs, where severe falls have a more significant social and economic impact [11]. Thus, some applications of the WGF algorithm may require adaptations to adequately address resource limitations and the specific needs of these countries [6]. Limited knowledge of the algorithm's applicability in LMIC populations hinders the development of targeted health policies for fall prevention. Recently, Lee et al. (2024) evaluated the predictive capacity of the WGF algorithm for fall prediction in community-dwelling older adults in Malaysia [12]. The authors used only the KQ-based version of the algorithm without exploring whether the isolated use of FH could also discriminate individuals at intermediate risk.

Therefore, new studies need to assess whether the different WGF approaches result in variations in fall risk prevalence among community-dwelling older adults to clarify a potential distinction between low- and intermediate-risk groups. Additionally, applying the WGF algorithm in the Brazilian context—a middle-income country—is crucial for accurately identifying risk levels, enabling more effective resource allocation and prevention strategies.

In this context, the objectives of the present study were: (1) to estimate the national and regional prevalence of fall risk among older Brazilian adults for each operational approach; and (2) to analyze differences in fall risk prevalence across geographic macroregions.

## Methods

### Study design and ethical aspects

This cross-sectional study used data from 7515 participants aged 60 years or older interviewed in the third wave of the Brazilian Longitudinal Study of Aging (ELSI-Brazil, 2023–2024). ELSI-Brazil is a nationally representative, household-based longitudinal survey conducted among individuals aged 50 years or older. The sample comprises residents from 70 municipalities across Brazil’s five macroregions. The sampling process was stratified by clusters across multiple stages, including primary sampling units (municipalities), census tracts, and households, to reflect the diversity of urban and rural areas across municipalities of varying sizes. Additional details about the sampling process and study design can be found in previous publications [13, 14]. Further information is also available on the study’s homepage (https://elsi.cpqrr.fiocruz.br/en/home-english/).

The Research Ethics Committee of the René Rachou Institute, Oswaldo Cruz Foundation—Minas Gerais, approved the ELSI-Brazil study (CAAE: 58766322.6.0000.5091). Before participating, all participants signed an informed consent form for each study procedure. A proxy respondent was used to answer the individual interview and provide written informed consent when necessary, which was duly documented.

### Outcome

The outcome was the fall risk classification proposed by the WFG, which categorizes participants into three risk levels (low, intermediate, and high) based on the FH or the KQ, integrated with the assessment of gait speed and the presence of severity markers. The FH was assessed with the following question: “In the last 12 months, have you had a fall?” (yes or no). The KQ involved the FH, the concern about falling, and instability during orthostatism. The concern about falling was assessed using the short version of the Falls Efficacy Scale-International (Short FES-I) [15]. The Short FES-I comprises seven items derived from the original FES-I, each item rated on a 4-point Likert scale from 1 (not at all concerned) to 4 (very concerned). Item scores are added to produce a total score between 7 and 28, with higher scores indicating more concern about falling. In this study, participants were categorized as having low concern (scores 7–10) or high concern (scores 11–28) based on cutoff points established by Delbaere et al. [16]. Instability during orthostatism was assessed using the tandem stance test, with instability defined as holding the position for less than 10 s. This test was used as a proxy for subjective stability [7].

**Fig. 1:**
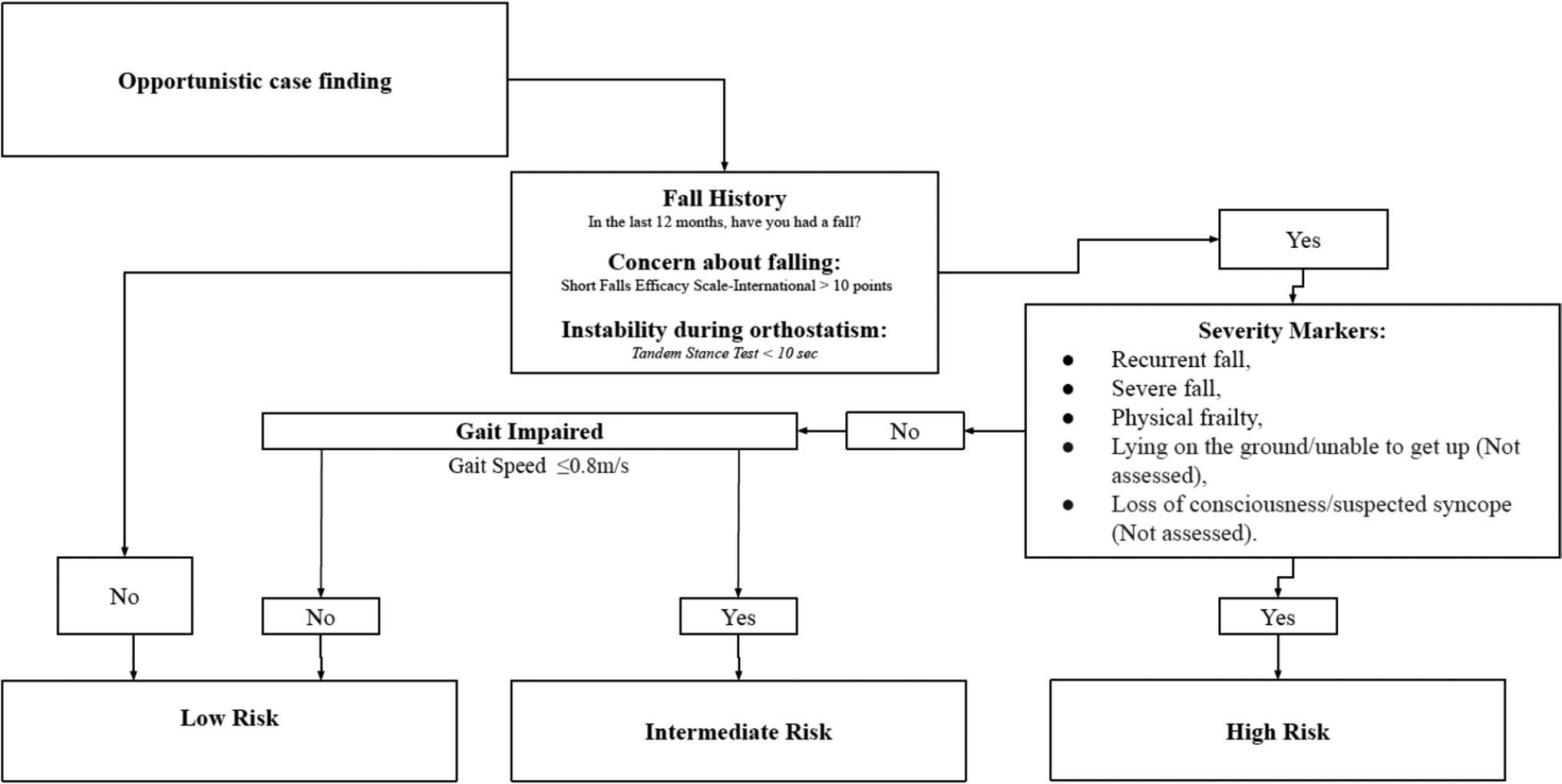
Conceptual framework for fall risk stratification, assessment, and management based on the World Guidelines for Falls Prevention, as applied to the ELSI-Brazil Study

The high-risk group included older adults with a positive criterion for the FH or KQ, both associated with the presence of severity markers. The intermediate-risk group included older adults with a positive criterion for the FH or KQ, without severity markers, but with low gait speed. The low-risk group included older adults with a positive criterion for the FH or KQ, without severity markers and low gait speed, or those without a positive criterion for the FH or KQ. Being positive for KQ means being positive on at least one criterion (Fig. 1).

The severity markers considered were at least one of the following: recurrent fall, severe fall, and physical frailty. A recurrent fall was defined as having suffered two or more falls in the last 12 months. A severe fall was defined as having suffered at least one fall in the last 12 months that resulted in a hip/femur or wrist/forearm fracture. Physical frailty was defined by the presence of three or more positive criteria based on the five criteria proposed by Fried et al. [17]: (1) Weight loss: self-reported weight loss of 4.5 kg or more in the last 3 months, without any intention or diet. (2) Exhaustion: frequencies greater than 3–4 days for any of the following questions: “In the last week, how often have you felt you could not handle your activities (started something, but could not finish it)?” and “In the last week, how often have your daily activities required a big effort from you to be performed?” (3) Muscle weakness: handgrip strength in the lower quintile, adjusted for sex and body mass index (BMI) quartiles. Strength was assessed in three attempts using a handheld dynamometer on the dominant upper limb, considering the best performance [18]. (4) Gait slowness: the quintile with the longest time spent walking 3 m at a usual pace, stratified by sex and height. (5) Low physical activity level: lower quintile of energy expenditure measured in kilocalories (kcals) per week, stratified by sex. The kcals expended over the past week on work activities, travel, leisure, sports, exercise, or household chores were recorded, accounting for intensity (light, moderate, or vigorous) and duration (minutes or hours). The criteria "lying on the ground/unable to get up" [7, 10, 12] and "loss of consciousness/suspected syncope" [7, 10], used as severity markers in the algorithm, were not considered in this study because there were no corresponding questions in the research questionnaire.

The gait speed test involved walking 3 m at the usual pace, in the usual footwear, and with an assistive device, if necessary. The test was performed twice, with no acceleration or deceleration distances, and a 1-min rest interval between measurements [19]. The average time of two attempts was used in the analysis. Gait speed was calculated as the distance (3 m) divided by the time taken to walk (s). We considered low gait speed when it was ≤ 0.8 m/s.

### Statistical analysis

Stata version 14.0 (https://www.stata.com) was used for data analysis. The effect of the sampling design and individual weights was incorporated into all analyses using the svy (survey) command. Prevalence rates (%) and 95% confidence intervals (95% CIs) were presented for each risk level in Brazil, by region, and age group. Adjusted prevalence ratios (PRs) and their respective 95% CIs were estimated using Poisson regression with robust variance, controlling for sex (female or male), age group (60–69, 70–79, or ≥ 80 years), years of schooling (< 8, 9–11, or ≥ 12 years), per capita household income in tertiles, and place of residence (urban or rural) to assess regional differences in the prevalence of fall risk. The South region was used as the reference category. Statistical significance was set at *p* < 0.05.

## Results

The third wave of the ELSI-Brazil study included individuals aged 50 years or older, totaling 10773 participants. Of these, 8571 participants were aged 60 years or older and were therefore eligible for the present analysis. Participants who did not complete the physical performance tests (*n* = 684), those who were bedridden or wheelchair users (*n* = 250), and individuals with missing information for one or more variables required for the fall risk algorithm (*n* = 122) were excluded, resulting in a final analytical sample of 7515 older adults. Additionally, the balance test variable presented 316 missing values; therefore, for the secondary classification analysis, the sample comprised 7199 participants.

The national prevalence was 82.2% (95% CI: 79.8; 84.3) for low fall risk, 7.8% (95% CI: 6.8; 9.1) for intermediate fall risk, and 10.0% (95% CI: 8.7; 11.4) for high fall risk, considering the FH alone. When using the KQ, the prevalence rates for low, intermediate, and high fall risk were 50.4% (95% CI: 46.5; 54.3), 34.6% (95% CI: 31.4; 37.7), and 15.0% (95% CI: 13.4; 16.6), respectively (Table 1). Description of prevalence estimates of fall risk for both screening approaches stratified by age group is presented in the supplementary material.

**Table 1:** National and regional prevalence of fall risk in community-dwelling older Brazilian adults. ELSI-Brazil, 2023–2024

	Prevalence of fall risk ^a^ % (95% CI)					
	Brazil	North region	Northeast region	Southeast region	South region	Central-West region
Fall History						
Low fall risk	82.2	80.4	78.7	81.9	85.1	89.9
	(79.8; 84.3)	(73.8; 85.7)	(73.6; 83.1)	(79.4; 84.2)	(78.5; 89.9)	(85.3; 93.2)
Intermediate fall risk	7.8	8.2	8.8	8.6	6.4	3.8
	(6.8; 9.1)	(5.3; 12.1)	(6.7; 11.5)	(7.2; 10.2)	(4.2; 9.5)	(1.9; 7.6)
High fall risk	10.0	11.4	12.5	9.5	8.5	6.3
	(8.7; 11.4)	(8.6; 15.1)	(9.8; 15.8)	(8.0; 11.2)	(5.5; 12.8)	(4.5; 8.7)
n total (unweighted)	7515	556	1985	3108	1097	769
Key Questions						
Low fall risk	50.4	50.0	44.8	46.7	65.0	59.9
	(46.5; 54.3)	(37.8; 62.1)	(38.5; 51.3)	(41.6; 51.8)	(57.9; 71.5)	(42.6; 75.0)
Intermediate fall risk	34.6	31.8	36.7	39.2	23.9	26.9
	(31.4; 37.7)	(24.0; 40.8)	(32.5; 41.1)	(35.0; 43.5)	(18.5; 30.2)	(13.3; 47.0)
High fall risk	15.0	18.2	18.5	14.1	11.1	13.2
	(13.4; 16.6)	(14.0; 23.3)	(15.1; 22.4)	(12.4; 16.1)	(7.5; 16.0)	(10.3; 16.7)
n total (unweighted)	7199	534	1896	2998	1054	717

CI = Confidence interval

The estimates considered the individual weights and the complex sample design

^a^Unadjusted prevalence

**Fig. 2:**
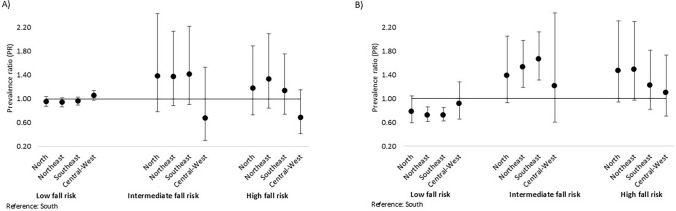
Regional differences in adjusted prevalence ratios for low, intermediate, and high fall risk among older Brazilian adults, **A** using fall history and **B** using the key questions. Poisson regression with robust variance was employed, adjusted for sex, age group, years of schooling, per capita household income in tertiles, and place of residence. The South region was used as the reference category. ELSI-Brazil, 2023–2024

Figure 2 presents the adjusted PRs of fall risk categories across Brazilian geographic macroregions based on the FH or the KQ. Considering only FH, no statistically significant differences across geographic macroregions in any fall risk category were observed (Fig. 2A). In contrast, when the adjusted models incorporated the KQ, regional differences emerged (Fig. 2B). Compared with the South region, older adults in the Northeast (PR = 0.72; 95% CI: 0.61–0.86) and Southeast (PR = 0.73; 95% CI: 0.62–0.85) had a significantly lower probability of being classified as low fall risk. No statistically significant differences were found for the North (PR = 0.78; 95% CI: 0.59–1.04) or Central-West (PR = 0.92; 95% CI: 0.66–1.28) regions. Additionally, individuals from the Northeast (PR = 1.53; 95% CI: 1.19–1.97) and Southeast (PR = 1.66; 95% CI: 1.31–2.11) had a higher probability of being classified as having intermediate fall risk than those living in the South. No statistically significant differences were found for the North (PR = 1.38; 95% CI: 0.93–2.04) or Central-West (PR = 1.21; 95% CI: 0.60–2.43) regions. Regarding high fall risk, no statistically significant regional differences were observed relative to the South region (Fig. 2B).

**Fig. 3:**
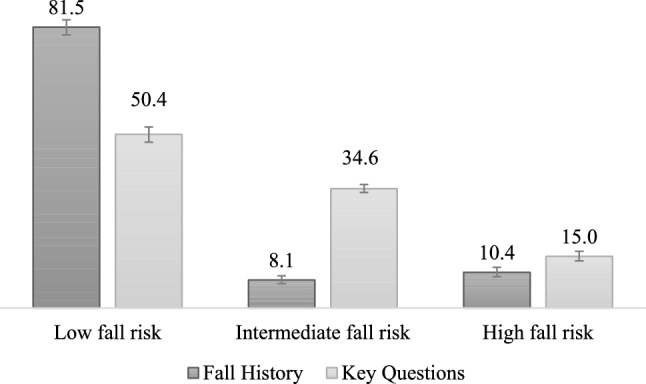
Distribution (%) of fall risk classification based on fall history and key questions *Note* Analyses presented in this figure were restricted to participants with complete data on both screening approaches

Figure 3 illustrates the distribution of fall risk categories according to two screening approaches: FH and KQ. When classification was based on FH, the majority of older adults were categorized as low fall risk [81.5% (95% CI: 79.2; 83.6)], with smaller proportions classified as intermediate [8.1% (95% CI: 7.0; 9.4)] and high fall risk [10.4% (95% CI: 9.1; 11.8)]. In contrast, the use of KQ resulted in a different distribution, with a lower proportion of individuals classified as low fall risk [50.4% (95% CI: 46.5; 54.3)] and higher proportions classified as intermediate [34.6% (95% CI: 31.5; 37.8)] and high fall risk [15.0% (95% CI: 13.4; 16.7)] than the FH approach.

## Discussion

The main findings of this study reveal differences in fall risk prevalence when using different WFG approaches. The national prevalence rates were 82.2% for low fall risk, 7.8% for intermediate fall risk, and 10.0% for high fall risk using FH alone. However, when using KQ, the prevalence rates were 50.4%, 34.6%, and 15.0% for low, intermediate, and high fall risk, respectively. Additionally, we found significant differences in fall risk prevalence among Brazilian geographic macroregions only when using the KQ approach.

Our results demonstrate that including KQ increased the proportion of older adults in the intermediate-risk group, aligning with the findings of van de Loo [7]. In that study, when using KQ, 61.3% of Dutch older adults were classified as low risk, 10.6% as intermediate risk, and 28.1% as high risk. Alternatively, when the algorithm was applied using the FH alone, the prevalence rates were 78.4% for low risk, 3.0% for intermediate risk, and 18.6% for high risk [7]. The authors concluded that the exclusive use of FH may underestimate the prevalence of intermediate fall risk. Identifying individuals in the intermediate-risk group could, in theory, help detect patients who could benefit preventively from exercise-based interventions, such as pre-frail older adults [7].

On the other hand, when comparing our findings with those of Lee et al. [12], differences emerge in the proportions of older adults assigned to each risk category. Using the KQ, Lee et al. [12] found that, in Malaysia, 79.4% of older adults were classified as low risk, 5.5% as intermediate risk, and 15.2% as high risk. The lower proportion of older adults in the intermediate-risk group in Malaysia may be attributed to sociodemographic and cultural factors, as well as to possible differences in the algorithm’s application. Lee et al. [12] used the Timed Up and Go (TUG) test as a substitute for gait speed. Studies that used TUG instead of gait speed also found lower prevalence rates of older adults in the intermediate fall risk group [8, 9, 12]. It is important to highlight that the algorithm recommends using gait speed as an essential predictor of fall risk, as the scientific literature demonstrates its strong association with functional capacity and fall risk in older adults. A previous umbrella review also concluded that gait speed is useful for predicting falls and should be considered as part of a comprehensive assessment in older adults [20]. The TUG can serve as an alternative measure, although evidence regarding its effectiveness in predicting falls is inconsistent. Studies have shown conflicting results about its ability to accurately identify individuals at risk of falling [6]. Therefore, these findings indicate that the prevalence of fall risk may vary depending on how the WFG algorithm is operationalized and the functional tests employed, underscoring the need for additional studies to assess the applicability of these tests across different populations.

In the present study, when using the FH alone for fall risk stratification and the gait speed to discriminate between low and intermediate-risk groups, prevalence rates of 82.2%, 7.8%, and 10.0% were observed for low, intermediate, and high fall risk, respectively. These findings are similar to the results of the study by Ragusa et al. [10], conducted with older adults in the USA, which also used the FH and gait speed as proposed by the WGF algorithm. The authors found prevalence rates of 82.0% for low risk, 0.6% for intermediate risk, and 17.3% for high risk. The main difference between the studies was the higher prevalence of intermediate risk in our sample, possibly due to specific population characteristics, including cultural or socioeconomic factors related to nationality.

In our study, the national prevalence of high fall risk was 10.0% with FH alone and 15.0% with KQ. Pimentel et al. [21] conducted a study using baseline data from ELSI-Brazil (2015–2016) to assess the prevalence of falls and their associated factors among older Brazilian adults residing in urban areas. The authors showed that 25.1% of participants had an FH. Unlike the present study, which assessed fall risk (FH or KQ + severity markers of falls and/or gait speed) in older adults residing in urban and rural areas, Pimentel et al. [21] focused exclusively on the FH of urban older adults. Thus, assessing fall risk rather than relying solely on FH offers a more comprehensive, preventive public health approach. Incorporating a fall risk approach might reduce fall incidence, improve the quality of life for older adults, and lower the costs of treating fall-related injuries. Therefore, it is crucial to distinguish between fall risk assessment and the mere presence of a positive FH, as they represent distinct dimensions.

Regarding geographic macroregions, we found that the prevalence of high-risk varied from 6.3% in the Central-West region, using the FH alone, to 18.5% in the Northeast region when considering KQ. No previous study has compared the prevalence of fall risk in the older population across the Brazilian geographic macroregions. Nonetheless, a prior meta-analysis showed that individuals in the Central-West region had a higher prevalence of falls than those in the South region [2]. The authors pointed out the scarcity of studies in the North region, with only one included, and emphasized the need for further investigations to provide more accurate estimates of fall prevalence. Moreover, most of the studies reviewed were performed in regions with higher population density, such as the South and Southeast, which accounted for about 80.0% of the estimates in the meta-analysis [2]. Regional differences in fall risk were not previously explored due to Brazil's substantial territorial extent, socioeconomic disparities, and distinct aging patterns across its regions.

In the study by Siqueira et al. [22], the prevalence of falls among older adults differed significantly across Brazilian geographic macroregions, varying from 18.6% to 30.0% in the North and Southeast regions, respectively. The lower prevalence observed in the North region may be related to its lower life expectancy. As the prevalence of falls tends to increase with age, regions with higher longevity, such as the Southeast, tend to have higher fall estimates. These findings indicate a possible association between life expectancy and fall risk. Factors such as cultural differences, lifestyle, and barriers to access to human and technological resources may also influence regional variations in fall prevalence rates [6].

This study has some limitations, so results should be interpreted with caution. First, the WFG fall risk stratification algorithm was slightly modified by omitting the components "lying on the ground/unable to get up" and "loss of consciousness/suspected syncope" due to the lack of this information in the ELSI-Brazil survey, which may have impacted the accuracy of the stratification, especially for older adults who experience more severe falls. Additionally, in our study, we assessed concern about falling using the Short FES-I, which provides a graded measure, whereas the original algorithm relies on a single self-reported question to capture this concern. Similarly, instability was operationalized using a balance-based proxy measure (tandem stance test), whereas the algorithm recommends self-reported instability during gait or orthostatism. These methodological differences are acknowledged as limitations and may have influenced the sensitivity of our results. Another limitation of our study is the exclusion of individuals who were bedridden, wheelchair users, or unable to complete physical performance tests. As these participants would likely have been classified as high risk based on interview-derived information (e.g., fall history or severity indicators), their exclusion may have led to an underestimation of the true national prevalence of high fall risk. On the other hand, this is the first study to stratify fall risk using the WFG in a nationally representative sample of older Brazilian adults. Our results are highly relevant, as the applicability of the WFG algorithm in LMICs remains underexplored [12].

## Conclusion

Given the lowest national prevalence of older adults classified as high fall risk (10%) and the most recent national estimates of the older population (32,113,490 people aged 60 years or older) [23], our findings suggest that approximately 3.5 million older Brazilian adults may be at high risk of falls. Our findings also highlighted regional disparities in fall risk, underscoring the need for context-specific interventions tailored to each region and territory to mitigate these inequalities.

## Supplementary Information

Below is the link to the electronic supplementary material.

Supplementary file1 (DOCX 16 KB)

## Data Availability

The data that support the findings of this study are available from the Brazilian Longitudinal Study of Aging (ELSIBrazil) website. Access to the data is subject to approval by the ELSI-Brazil data access committee and can be requested through the study’s official website (https://elsi.cpqrr.fiocruz.br/en/home-english/register/).
