# Supplementary material for: Fall risk assessment using the World Guidelines for Falls Prevention Algorithm: Evidence from the ELSI-Brazil study
Source: Eur Geriatr Med. 2026 Apr 1;17(3):1459–66. doi: 10.1007/s41999-026-01446-6 (PMC13309500; doi:10.1007/s41999-026-01446-6)
Supplement: Supplementary file 1 — Supplementary file1 (DOCX 16 KB) [file 41999_2026_1446_MOESM1_ESM.docx]

**Supplementary Table** - Prevalence of fall risk in community-dwelling older Brazilian adults by age group. ELSI-Brazil, 2023-2024.

|  | **Prevalence of fall risk**  **% (95%CI)** | | |
| --- | --- | --- | --- |
|  | 60–69 years | 70–79 years | ≥80 years |
| **Low fall risk** | 84.7 | 80.8 | 73.4 |
|  | (82.2; 86.9) | (78.2; 83.2) | (69.5; 76.9) |
| **Intermediate fall risk** | 7.2 | 9.0 | 8.1 |
|  | (5.9; 8.7) | (7.5; 10.7) | (6.1; 10.6) |
| **High fall risk** | 8.1 | 10.2 | 18.5 |
|  | (6.8; 9.6) | (8.7; 11.9) | (15.8; 21.7) |
| **n total (unweighted)** | 3,667 | 2,574 | 1,274 |
| **Low fall risk** | 57.9 | 44.8 | 28.2 |
|  | (53.6; 62.1) | (40.5; 49.2) | (23.6; 33.3) |
| **Intermediate fall risk** | 30.7 | 39.4 | 42.1 |
|  | (27.1; 34.4) | (35.7; 43.1) | (37.9; 46.5) |
| **High fall risk** | 11.4 | 15.8 | 29.7 |
|  | (10.0; 13.0) | (13.7; 18.2) | (26.1; 33.5) |
| **n total (unweighted)** | 3,524 | 2,480 | 1,195 |

CI = confidence interval.

The estimates considered the individual weights and the complex sample design.
